# Deciphering the Genetic Basis of Root and Biomass Traits in Rapeseed (*Brassica napus* L.) through the Integration of GWAS and RNA-Seq under Nitrogen Stress

**DOI:** 10.3390/ijms23147958

**Published:** 2022-07-19

**Authors:** Nazir Ahmad, Bin Su, Sani Ibrahim, Lieqiong Kuang, Ze Tian, Xinfa Wang, Hanzhong Wang, Xiaoling Dun

**Affiliations:** 1 Oil Crops Research Institute of the Chinese Academy of Agricultural Sciences/Key Laboratory of Biology and Genetic Improvement of Oil Crops, Ministry of Agriculture and Rural Affairs, Wuhan 430062, China; nazir_aup@yahoo.com (N.A.); su13297095753@163.com (B.S.); sibrahim.bot@buk.edu.ng (S.I.); kuanglieqiong@163.com (L.K.); tianze0825@163.com (Z.T.); wangxinfa@caas.cn (X.W.); 2 Department of Plant Biology, Faculty of Life Sciences, College of Physical and Pharmaceutical Sciences, Bayero University, P.M.B. 3011, Kano 700006, Nigeria; 3 Hubei Hongshan Laboratory, Wuhan 430070, China

**Keywords:** rapeseed, root and biomass traits, nitrogen stress, GWAS, RNA sequencing

## Abstract

An excellent root system is responsible for crops with high nitrogen-use efficiency (NUE). The current study evaluated the natural variations in 13 root- and biomass-related traits under a low nitrogen (LN) treatment in a rapeseed association panel. The studied traits exhibited significant phenotypic differences with heritabilities ranging from 0.53 to 0.66, and most of the traits showed significant correlations with each other. The genome-wide association study (GWAS) found 51 significant and 30 suggestive trait–SNP associations that integrated into 14 valid quantitative trait loci (QTL) clusters and explained 5.7–21.2% phenotypic variance. In addition, RNA sequencing was performed at two time points to examine the differential expression of genes (DEGs) between high and low NUE lines. In total, 245, 540, and 399 DEGs were identified as LN stress-specific, high nitrogen (HN) condition-specific, and HNLN common DEGs, respectively. An integrated analysis of GWAS, weighted gene co-expression network, and DEGs revealed 16 genes involved in rapeseed root development under LN stress. Previous studies have reported that the homologs of seven out of sixteen potential genes control root growth and NUE. These findings revealed the genetic basis underlying nitrogen stress and provided worthwhile SNPs/genes information for the genetic improvement of NUE in rapeseed.

## 1. Introduction

Nitrogen (N) is one of the most important macronutrients required for plant growth and development. It is the basic component of proteins, nucleic acids, chlorophyll, and several hormones [1,2]. Although global agricultural N consumption has increased sevenfold in the last half-century, most crops utilize only 30–40% of the supplied N [3,4]. A high N fertilizer application causes serious environmental problems, such as water eutrophication, acid rain, and soil acidification [5]. To ensure the sustainability of agriculture, it is imperative to breed crop varieties with a higher nitrogen use efficiency (NUE) [6].

Rapeseed (*Brassica napus* L.) is the third-largest oil crop in the world, following soybeans and palm. However, while absorbing a significant amount of N from the soil, rapeseed is generally regarded as a low NUE crop, with seed yield per unit N applied about half that of other cereals [7]. Therefore, addressing the genetic architecture of low nitrogen stress tolerance and boosting rapeseed’s NUE is vital for the rapeseed’s economic competitiveness [8,9]. Root system architecture (RSA) plays a critical role in N acquisition, both in terms of absorption capacity and soil exploration potential. A deeper understanding of how the RSA adapts to N availability seems to be a potential lever for optimizing N acquisition [10,11,12]. As a primary step, determining the key traits underpinning genotype variability and RSA adaptability to N availability is required. For example, in most elite cultivars, increasing the root-to-shoot ratio increases the uptake of N from the deep soil, or the longer roots provide optimum root nutrient storage in shoots by taking advantage of the soil’s spatial characteristics [13,14,15]. Furthermore, the genetic improvement of root morphological traits was reported to affect crop yield [16]. Rice yields have been shown to increase under drought stress when *DRO1*, a QTL associated with both root depth and root development angle, is expressed at a higher level [17]. The *big root biomass* (*BRB*) gene was reported previously to affect shoot traits and seed yield in sesame [18]. In rapeseed, coarse root length promotes soil exploration and phosphorus uptake, which increases seed yield [19]. As a result, optimizing root-related parameters could be a potential strategy for promoting the development of cultivars with high NUE and yield.

Genome-wide association study (GWAS) is an effective method for identifying loci/genes associated with complex traits in a genetically diverse crop population, including root architectural traits in rice, wheat, soybean, maize, rapeseed, etc. [20,21,22,23,24,25,26]. Transcriptome analysis has proven to be valuable for identifying candidate genes, especially differentially expressed genes (DEGs), between samples with contrasting traits. The weighted gene co-expression network analysis (WGCNA) method was designed to explore the system-level functionality of the transcriptome and is widely used in plants to identify gene modules related to the uncovering of potential transcriptional regulation [27,28]. The combination of GWAS, transcriptome sequencing, and/or WGCNA has proven to be a quick and effective strategy for detecting major candidate genes regulating complex traits. In response to cadmium stress, three hub-genes (*OsHSP*, *OsHSFC2A*, and *OsDJA5*) were identified through RNA sequencing and WGCNA in rice [29]. Through the integration of GWAS, WGCNA, and differential expression analysis, four and eight important candidate genes related to root growth in *B. napus* were identified during the persistent and specific stages, respectively [30].

Due to the hidden feature of roots, the main challenge in studying root traits is developing robust phenotypic evaluation methods. Different artificial systems have been used to evaluate root traits, including sand, germination paper, and hydroponic-based cultures [31]. Hydroponic culture with digital imaging could quickly and precisely detect a variety of root traits in large populations and has been used to examine variations in root architecture among different crops, including rice, wheat, maize, soybean, and rapeseed [25,30,32,33,34,35]. In the present study, 13 RSA traits were investigated in hydroponic culture under control and low nitrogen (LN) treatments in an association panel of 327 *B. napus* cultivars, which was genotyped by the 50 K *Brassica* Infinium SNP array [25]. RNA-seq was performed in the high and low nitrogen efficient groups at two developmental stages to determine the expression levels of candidate genes. The goals of this study were to examine the phenotypic variations of 13 root and biomass traits under both control and LN treatments within a rapeseed association panel, to identify significant SNPs associated with root and biomass traits related to NUE, and to use a GWAS and RNA-seq approach to determine potential candidate genes associated with root response to LN stress.

## 2. Results

### 2.1. Phenotypic Analysis of Root and Shoot Biomass Traits under the LN Stress

A total of 13 root and shoot biomass traits across 327 accessions in two treatment conditions (control and LN stress) were evaluated under hydroponics, including five root morphological traits (MT) and eight biomass traits (BT) (Table 1). 

For the investigated traits in the association panel, extensive variations were observed among genotypes in control and LN-treated conditions (Table 2). The coefficient of variation (CV) of the 13 traits ranged from 14.5–45.6% and 12.8–31.9%, respectively (Table 2). In both treatment conditions, moderate to high broad-sense heritability was observed for all the studied traits, with the value of 0.53 to 0.70. Overall, these results showed that all 13 traits were inherited in a stable manner under hydroponics in both control and LN stress conditions. LN treatment affected all investigated root and biomass traits compared with the control (Table 2). On average, LN treatment significantly increased length, number, and area of roots in the association population, resulting in an increase in RFW (6.4%) and RDW (14.2%). However, LN treatment significantly reduced the formation of aboveground biomass, with SFW and SDW decreased by 80.3% and 24.7%, respectively. Therefore, the root–shoot ratio increased significantly under LN treatment, RSRF and RSRD by 48.9% and 33.8%, respectively.

**Figure 1:**
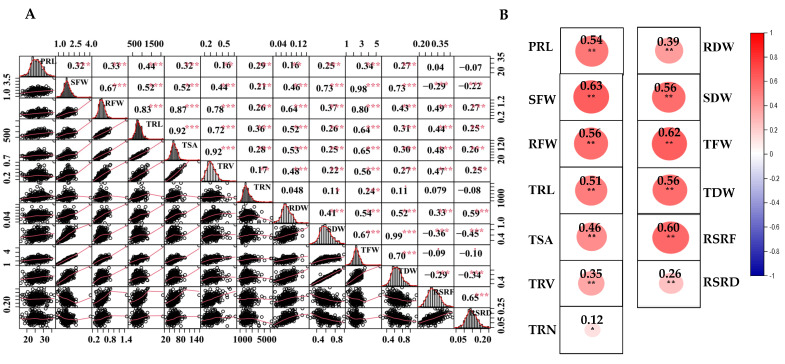
Correlation analysis of the investigated traits. (**A**) Correlations of studied traits under low nitrogen stress. Frequency distribution for each trait was displayed on the diagonal. The upper and lower parts represent the correlation coefficient and scatter plots between two diagonal traits, respectively. (**B**) Correlations of each investigated trait between control and low nitrogen stress. Red and blue indicate positive and negative correlations, respectively. ***, ** and * denote significance at the 0.1%, 1% and 5% levels of probability, respectively.

Consistent with the correlations under the control condition [30], SFW were positively and significantly correlated with RFW under LN stress (r = 0.67, *p* < 0.01). Between aboveground biomass and root morphological traits, SFW displayed the highest correlations with TRL (r = 0.52, *p* < 0.01), indicating that root morphology contributed significantly to the formation of aboveground biomass. Negative correlations were also observed between shoot biomass traits and root–shoot ratios, with the values of −0.29 between SFW and RSRF and −0.45 between SDW and RSRD, respectively (Figure 1A). Furthermore, root and biomass traits showed positive and significant correlations between CK and LN stress treatment, and the correlation of SFW (r = 0.63, *p* < 0.01) was the highest, while TRN (r = 0.12, *p* < 0.05) had the lowest correlation (Figure 1B). It further illustrated that it is essential to consider multiple inter-related traits to comprehensively assess nitrogen efficiency concerning RSA traits. In addition, the frequency distributions for 13 parameters under LN stress were nearly continuous and normal, demonstrating that the examined accessions were appropriate for subsequent association study (Figure 1A).

### 2.2. Marker–Trait Association Analysis for Root and Biomass Traits under LN Stress

**Figure 2:**
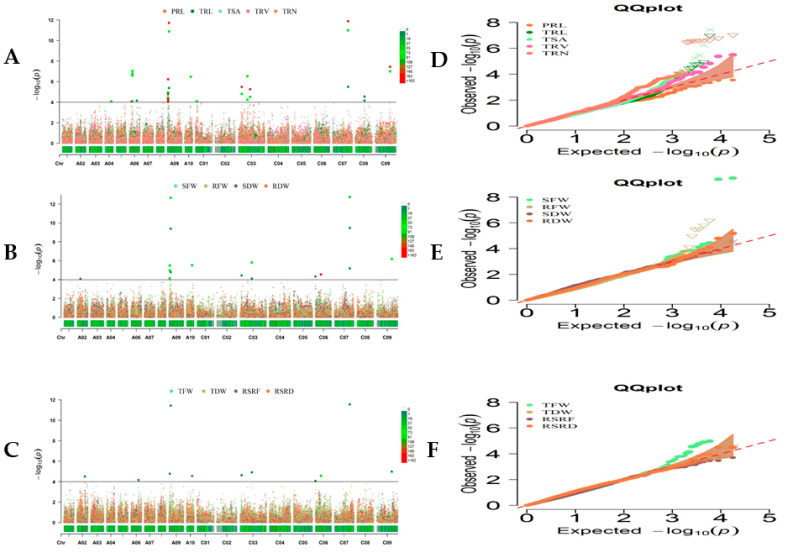
(**A**–**C**) Manhattan plots of the phenotype–genotype association analysis for 13 root and shoot biomass traits of *B. napus* by MLM with BLUE values. The x-axis displays the chromosome label, and the y-axis displays −log_10_ (*p*-value). The solid gray line shows significant associations between SNPs and phenotype value with threshold level of *p*-value (−log_10_ 1/20,131 = 4.30 × 10^−5^). The color dots above the threshold values indicate the significant SNPs for root and shoot biomass traits. (**D**–**F**) QQ plots represent MLM analysis of the above 13 traits.

The association panel was genotyped using the Brassica 50 K Illumina Infinium SNP array containing 45,708 SNPs. As a result of SNP filtering, 20,131 SNP markers were used to further identify trait–SNP associations [25]. This study only performed genome-wide association analyses (GWAS) with BLUE values from three trials under LN stress (Figure 2A–F), since the results of association analysis under normal condition were shown in the previous study [30]. We grouped SNPs with close proximity (within 1 MB) and an LD r^2^ > 0.2 together, since they were found to be a part of the same QTL [36].

To avoid missing SNPs due to the complex nature of RSA traits and the strict criteria of MLM, we defined suggestive trait–SNP associations (3.50 < −log_10_ *p* ≤ −log_10_ 1/20,131). This resulted in 51 significant trait–SNP associations (−sslog_10_ > 4.30, −log_10_ 1/20,131), with 24 significant SNP markers and 30 suggestive trait–SNP associations integrated into 14 valid QTL clusters (Table 3 and Table S1), most of which included at least two investigated root and biomass-related traits. Genetic variation explained by these QTL clusters varied from 5.7 to 21.2%. These QTLs were detected for 11 RSA traits, except PRL and RSRF (Table 3). The highest number of loci were identified on A09 and C03, containing 28 and 18 loci (Table S1).

Since root and biomass traits have exhibited considerable and strong correlations, several pleiotropic genetic loci were identified, including QTL clusters RT-A09-1, RT-A09-2, RT-A10-1, RT-C03-4, RT-C07-1, and RT-C09-1, which affected both root development and aboveground biomass formation. In particular, the SNP seq-new-rs41996 in the QTL cluster RT-A09-2 was associated with both BT and RMT (RFW, TFW, SFW, RDW, TRL, TSA, and TRV), with the highest phenotypic contribution (R^2^) of 21.2% for RFW. Similarly, the SNP seq-new-rs46512 loci were also detected as pleiotropic on RT-C07-1 for RFW, TSA, TFW, TRL, SFW, TRV, and RDW, with the highest R^2^ of 20.4% for RFW (Table S1). After validation, these identified loci simultaneously influencing root and shoot biomass traits could be potential loci for marker-assisted breeding.

### 2.3. Differentially Expressed Genes (DEGs) between High and Low Nitrogen Efficient Group

**Figure 3:**
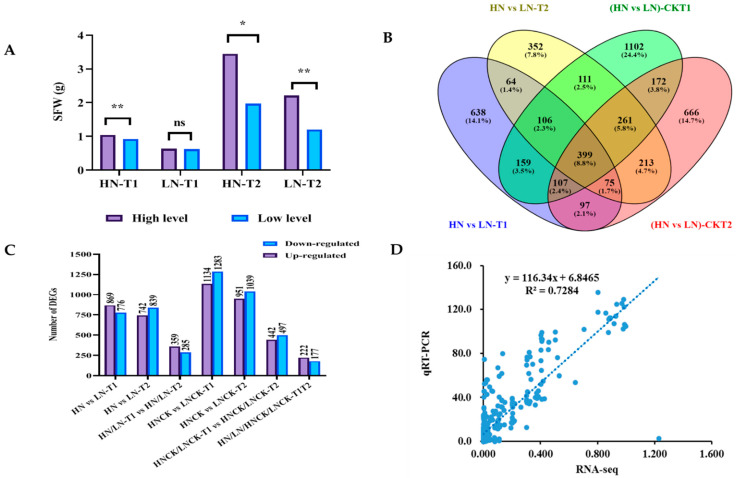
Differential gene expression analysis. (**A**) Phenotypic performance of two groups (HN and LN) at T1 and T2. (**B**) Venn diagram of the DEGs in the selected groups. (**C**) Up- and downregulated DEGs in different groups. (**D**) Correlation between qRT-PCR and RNA-seq data. ** and * denote significance at the 1% and 5% levels of probability, respectively. ns, not significant.

According to further phenotypic investigation, root tissues of 10 lines with extremely high SFW and 10 lines with extremely low SFW were selected as a high nitrogen-efficient group (HN group) and low nitrogen-efficient group (LN group), respectively, at two developmental points, T1 (7 days after transplantation) and T2 (14 days after transplantation) under both control and LN stress for RNA sequencing analysis (Figure 3A). Consequently, 24 libraries, including three biological replicates of the HN and LN groups under the low nitrogen stress, and HNCK and LNCK groups under the control condition, at T1 and T2 time points were generated. The total, mapped, and unique mapped reads to the reference *B. napus* genome are shown in Table S2. After filtering and trimming, the Illumina RNA-seq analysis yielded 1,153,760,000 clean reads. The average guanine–cytosine (GC) content was 46.97%, and all of the Phred quality scores (Q30) were above 94.35%. According to the principal component analysis (PCA) and correlation analysis based on gene expression levels, the correlation between individuals within the same groups was greater than the correlation between individuals within different groups (Figure S1A,B), indicating that the three biological repeats used in the experimental design were sufficiently accurate.

Using a pairwise approach, we first identified the DEGs of the HN and LN groups at T1 and T2 under control and LN stress, respectively, including HN/LN-T1 vs. HN/LN-T2 and HNCK/LNCK-T1 vs. HNCK/LNCK-T2. Then, the common DEGs under both control and LN stress conditions were identified between these two categories (HN/LN-T1/T2 vs. HNCK/LNCK-T1/T2) (Figure 3B). The DEGs between these groups were determined using a false discovery rate (FDR) ≤ 0.05, and an absolute value of |log2 (fold change)| was used as the threshold. In HN/LN-T1 vs. HN/LN-T2, 644 DEGs were identified, including 359 upregulated and 285 downregulated DEGs (Figure 3C). Similarly, in group HNCK/LNCK-T1 vs. HNCK/LNCK-T2, 939 DEGs were identified (442 upregulated and 497 downregulated). Furthermore, 399 DEGs (222 upregulated and 177 downregulated) were regarded as common DEGs for HN/LN/HNCK/LNCK-T1/T2 (Figure 3B,C). This meant that there were 245 DEGs specific to the HN/LN group under the LN stress condition, and 540 DEGs were specific under the control condition (Table S3).

Details of DEGs, their full names and FPKM values in each group, and corresponding description information are presented in Table S3. A heatmap was constructed using normalized FPKM values ranging from −1 to 1 to classify high/low N-specific DEGs based on expression profile similarity and diversity (Figure S2). The heatmap clearly exhibited the upregulated and downregulated clusters for the gene expression patterns of DEGs. Furthermore, qRT-PCR for 12 DEGs in all of the samples was strikingly similar to the RNA-Seq data, showing that the RNA-Seq data were accurate (Figure 3D).

### 2.4. Functional Classification of DEGs Involved in High and Low Nitrogen Efficiency

**Figure 4:**
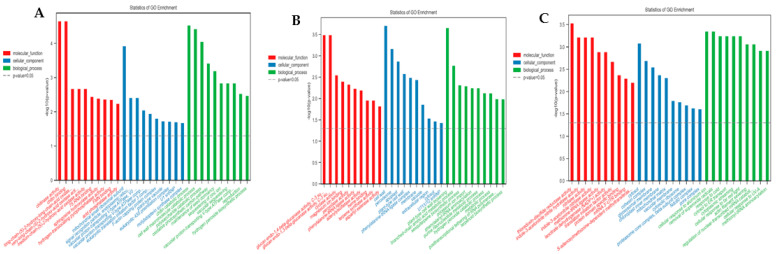
Gene ontology (GO) analysis of differentially expressed genes. (**A**–**C**) GO terms correspond to HN/LN group, HNCK/LNCK group, and HNLN/HNCK.LNCK group, respectively. Y-axis is −log_10_ (*p*-value). The relevant *p*-value decreases as the bar chart height increases. Red, blue, and green colors correspond to molecular function, cellular component, and biological process.

To further determine the functional significance of the DEGs in each group, gene ontology (GO) classifications were performed. In total, 245 specific DEGs under the LN stress condition, 540 specific DEGs under the control condition, and 399 common under both conditions were significantly assigned to 174, 128, and 159 GO terms, respectively (Table S4). Interestingly, all three groups of DEGs were enriched in different pathways. For LN-stress-specific DEGs, significant GO terms in molecular function were chitinase activity, chitin binding, medium-chain-(S)-2-hydroxy-acid oxidase activity, ATPase binding, and nutrient reservoir activity; in the cellular component category were mitochondrial small ribosomal subunit, signal recognition particle, and vacuolar proton-transporting V-type ATPase activity; in the biological function category were chitin catabolic process, cell wall macromolecule catabolic process and polysaccharide catabolic process, oxidative photosynthetic carbon pathway, maintenance of root meristem identity, and cellular response to reactive oxygen species (Figure 4A). 

According to the GO classification of the specific DEGs under the control condition, significant terms of molecular function were glucan endo-1-4-beta-glucanase activity, glucan endo-1-3-beta-glucanase activity, cyclase activity, and carbohydrate binding; the over-represented terms in the cellular function category were cell wall, apoplast, followed by peroxisome; in the biological function category, plant-type cell wall loosening, branched-chain amino acid metabolic process, sesquiterpene biosynthetic process, cell wall modification, negative regulation of cell division, reactive oxygen species metabolic process, maintenance of root meristem identity, and glucose metabolic process were the most over-represented terms (Figure 4B). 

For the common DEGs under both LN stress and control conditions, the significant GO terms of molecular function were thioredoxin-disulfide reductase activity, indole-3-acetonitrile nitrile hydratase activity, followed by nitrilase activity; the over-represented terms in the cellular function category were cytosol, cohesion complex, and glyoxysomal membrane, while, in the biological function category, the significant GO terms were cellular response to aluminum ion, removal of superoxide radicals followed by response to cold, NADP metabolic process, glutamate metabolic process, root hair elongation, regulation of cell shape, photosynthesis, nitrogen compound metabolic process, and cellular response to gravity (Figure 4C). 

By analyzing the common enrichment pathway of these three DEG groups of differential genes, it was found that 14 significant pathways composed of 35 DEGs were identified as common pathways for HN/LN efficiency. Most of these pathways were related to hydrogen-translocating pyrophosphatase activity (GO:0009678), acid phosphatase activity (GO: 0003993), translation initiation factor activity (GO: 0003743), magnesium ion binding (GO: 0000287), 3-isopropylmalate dehydrogenase activity (GO: 0003862), oxidoreductase activity (GO: 0016702), chloroplast envelope (GO: 0009941), maintenance of root meristem identity (GO: 0010078), cellular response to reactive oxygen species (GO: 0034614), fatty acid alpha-oxidation (GO: 0001561), cellular response to aluminum ion (GO: 0071275), (R)-2-hydroxy-alpha-linolenic acid biosynthetic process (GO: 1902609), and translational initiation (GO: 0006413) (Table S4). Thus, the regulation of these genes might play an important role in the N-efficient utilization in *B. napus*.

### 2.5. Gene Co-Expression Network Construction and Analysis (WGCNA)

**Figure 5:**
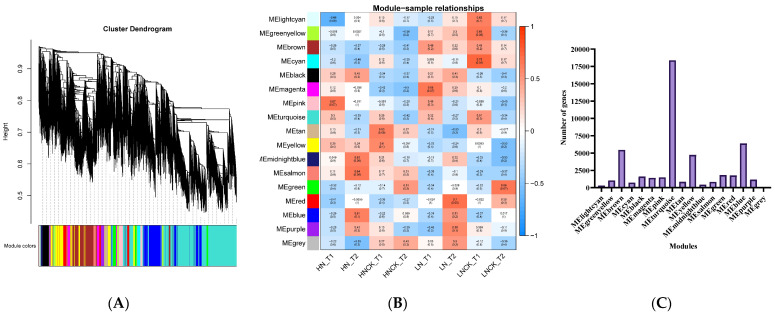
WGCNA of gene expression matrix. (**A**) Gene-based co-expression network analysis dendrogram. (**B**) Module–sample association; each row represents a module labeled with the same color as in (**A**), and each column represents a sample. (**C**) Overview of identified genes corresponds to each module.

In order to investigate the gene regulatory network during LN stress, WGCNA was used to determine co-expression gene modules from 83,232 identified expressed genes with *p* > 0.05. The dendrogram revealed a total of 17 modules based on gene correlations (Figure 5A), and the relationships between modules and samples are depicted in Figure 5B. In total, 48,385 genes were identified to be involved in these 17 modules, ranging from 46 in the “MEgrey” module to 18,376 in the “MEturquoise” module (Figure 5C). The MEpink, MEsalmon, MEtan, MEmagenta, MEred, and MEcyan modules were highly correlated with HN-T1, HN-T2, HNCK-T1, LN-T1, LN-T2, and LNCK-T1, respectively. MEgreen modules were found to be highly correlated with HNCK-T2 and LNCK-T2. In addition, the MEsalmon module showed a consistent correlation with all samples of the high nitrogen-efficient group (HN-T1, HN-T2, HNCK-T1, and HNCK-T2). Likewise, the MEbrown module revealed a high correlation with all samples of the low nitrogen-efficient group (LN-T1, LN-T2, LNCK-T1, and LNCK-T2) (Figure 5B). The heatmaps revealed that the genes contained within a single module were significantly expressed in samples that were strongly correlated with the module (Figure S3).

The GO and KEGG analysis suggested that the significantly enriched GO terms of genes in the MEsalmon module were related to mRNA processing, meristem development, NADPH-hemoprotein reductase activity, cytokinin biosynthetic process and response to oxidative stress, NADH pyrophosphatase activity, and root hair elongation (Table S5). Meanwhile, pyruvate metabolism, glycolysis, and oxidative phosphorylation pathways were significantly enriched in the “MEsalmon” module by KEGG pathway enrichment analysis (Table S6). Furthermore, significant GO terms in the “MEbrown” module were mRNA binding, gibberellin-mediated signaling pathway, cell communication, NAD metabolic process, regulation of carbon utilization, NADP biosynthetic process, and regulation of auxin-mediated signaling pathway (Table S5). The most enriched KEGG pathway in the “MEbrown” module was RNA transport, nicotinate, and nicotinamide metabolism, lysine biosynthesis, and N-Glycan biosynthesis aminoacyl-tRNA biosynthesis (Table S6). These important pathways played a crucial role in the nitrogen metabolism and assimilation process.

### 2.6. Candidate Genes’ Prediction and Prioritization by Integrating GWAS, DEGs, and WGCNA

**Figure 6:**
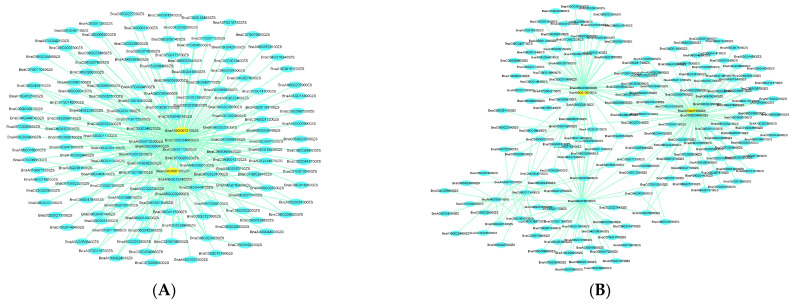
Network of genes in salmon and brown modules. (**A**,**B**) Correlation of networks in salmon and brown modules, respectively. Yellow color in the network indicates the candidate genes overlapped by GWAS and WGCNA.

Genes within 300 kb upstream and downstream of significant lead SNPs associated with each trait were revealed using the decay of the LD approach [37,38]. As a result, GWAS results revealed a total of 1378 genes around each peak SNP from 14 QTL clusters within the 300 kb region (up and down) (Table S7). The substantial and consistent correlation of WGCNA genes with each module allowed us to explore four potential genes from GWAS and WGCNA overlapped genes (Figure 6A,B). Among the four candidate genes, two genes with high and consistent correlation to the MEsalmon and two with MEbrown modules were highly expressed at all stages of the high nitrogen-efficient group and low nitrogen-efficient group, respectively (Table 4). Furthermore, we identified 12 genes simultaneously detected as common candidate genes by integrating GWAS and DEGs (Table 4). Within these 16 genes, we identified some potential candidate genes related to nitrogen use efficiency, nitrogen utilization, assimilation, and root growth and development. These findings assessed the efficiency of an approach for screening candidate genes that integrated GWAS, WGCNA, and differential expression analysis. 

## 3. Discussion

Nitrogen stress is a major limiting factor for crop production worldwide, and plant RSA is of great significance in nutrient stress tolerance [39]. In recent years, the use of RSA is predicted to result in a “second green revolution” in agriculture [13,40]. Different phenotyping methodologies for early crops’ RSA screening were used, expecting that genotypes with diverse root architecture at the seedling stage would respond similarly at the adult stage when water and/or nutrients became limited for grain yield [12]. In this study, we studied root and shoot behaviors at the seedling stage under N-limited conditions in a modified hydroponics growth system, which was deemed a valid way to examine root system changes compared to field conditions [41]. The significant variations were observed for different root and biomass traits among the genotypes of the association panel due to their diverse genetic background and wide geographical distributions. In agreement with the previous studies [26,42], seedlings grown under LN conditions showed reduced SFW and TFW but increased RSRF, SDW, and RSRD than seedlings grown under control (CK) conditions. This finding shows that N-deficient plants transport more carbon in order to promote root development and, hence, mine the substrate for more nitrogen [12]. Greater TRL, TRV, TSA, and TRN indicate an increased ability to acquire more N from the nutrient solution. Under control and low-N conditions, the broad-sense heritability of the examined traits was moderate to high (0.55–0.72) and (0.53–0.66), respectively (Table 2), indicating that these root and biomass parameters are more genetically governed and, thus, more responsive to genetic improvement. Similar findings were reported by other researchers [26,43].

Correlations between root and biomass traits demonstrate the balance of root and shoot organs and resource partitioning between above- and below-ground plant tissues [44]. In the present study, a high correlation between shoot and root weights observed might be due to the supply of nutrients from roots to the shoot parts, as reported in rapeseed [25,26,30,43,45]. Furthermore, the strong correlation between root and biomass traits aids in successful soil exploration by intercepting nutrients and communicating stress signals [46]. Understanding the mechanisms underlying root-related traits in crops may be an effective strategy for developing high-quality root cultivars via marker-assisted selection [41]. Based on GWAS results, 81 trait–SNP associations (51 significant and 30 suggestive) were identified that were integrated into 14 valid QTL clusters (Table 3, Table S1). This revealed the complex genetic control of root and biomass traits at an early stage of crop growth. In addition, some pleiotropic QTLs were found (Table S1), revealing that different traits from specific QTLs may be addressed separately to increase RSA in rapeseed [47].

Transcriptome analysis is a robust approach that helps the identification of differentially expressed genes with their expression level and regulatory mechanisms [48]. The present study identified 245 and 540 DEGs specific to HN/LN and HNCK/LNCK, respectively, while 399 DEGs were considered common DEGs for HN/LN/HNCK/LNCK-T1T2 (Table S3, Figure 3B). DEGs associated with significant GO terms might have a critical role in root growth and nitrogen efficiency/stress tolerance. For example, BnaC02G0384400ZS (AtGLO4) associated with GO-term medium-chain-(S)-2-hydroxy-acid oxidase activity has been reported to play a crucial role in carbon and nitrogen metabolism [49]. BnaC06G0457600ZS (AtGLYK), corresponding to the oxidative photosynthetic carbon pathway, has been recently reported to regulate nitrogen assimilation [50]. Similarly, BnaC02G0071500ZS (AtNLA), associated with GO term glucan endo-1-4-beta-glucanase activity, has been reported to play a key role in nitrogen remobilization and promote root elongation under nitrogen stress [51,52]. BnaC02G0065900ZS (AtGMII), associated with carbohydrate binding, has been reported to play a key role in root growth and development [53]. These findings indicate that DEGs encoding several metabolic, regulatory, signaling, and structural proteins involved in arginine and proline metabolism, galactose metabolism, and tryptophan metabolism may be primarily responsible for the differences in nitrogen use efficiency between these groups [54].

The integration of GWAS, WGCNA, and differential expression analysis has already been used to identify candidate genes in many crops [55]. We identified 12 and 4 candidate genes related to root growth and nitrogen stress by integrating GWAS, WGCNA, and DEGs. For example, BnaA09g04260D, a major hub gene in the MEsalmon module and located in the region of the QTL cluster qcA09-1, encodes a major facilitator superfamily protein, whose homologous AtSTP13 had a potential role in root growth responses and nitrogen uptake under nitrogen-starved conditions [56,57,58]. Another hub gene in the MEsalmon module and the qcA09-1 region, BnaA09g05270D, has been reported to have a crucial role in regulating root gravitropism and elongation against various environmental stresses [59,60]. An overlapped candidate gene between the MEbrown module and qcA09-2, BnaA09g08440D, a member of the NPY gene family (AtNPY1), had a crucial role in root gravitropism in *A.thaliana* [61]. Four potential candidate genes out of twelve were identified through the integration of GWAS, and DEGs have been reported to function as central regulators in root development and nitrogen stress. BnaA10g16560D (AtGSR2), encoding glutamine synthetase, has been reported to function in nitrogen assimilation, and thus improve nitrogen use efficiency [62,63]. BnaA10g19550D (AtLAZY1/AtANR1), an important candidate gene identified in RT-A10-2, has a crucial role in root gravitropism and nitrate regulation of root development [64,65]. BnaA10g19700D (AtXTH5) encoding endoxyloglucan transferase has been reported to regulate root cap during nitrogen stress [66]. Another important gene, BnaC07g30400D (AtSLAH3), plays a potential role in nitrogen uptake and assimilation during nitrogen-deprived conditions [67,68,69].

The aforementioned results revealed that these candidate genes played an important role in root growth and nitrogen utilization efficiency. A better understanding of nitrogen stress tolerance and root growth was acquired by identifying potential associated SNPs and promising candidate genes of nitrogen stress tolerance, which will serve a crucial role in the elite’s rapeseed breeding programs. However, further research based on these putative candidate genes will comprehensively elucidate the significance of these genes in rapeseed NUE, root development, and growth.

## 4. Materials and Methods

### 4.1. Plant Materials and Growth Conditions

Based on the Rapeseed Research Network in China, 327 *B. napus* lines were used in this study, including 191 semi-winter (population 1; P1), 34 winter (P2), and 102 spring accessions (P3). A total of 327 rapeseed germplasm accessions were studied, with 222 from China’s Yangtze River, 52 from other places/unknown origins, 23 from northwestern China, 16 from Europe, and 14 from Australia. All the accessions were strictly self-crossed.

The root-related traits of 327 *B. napus* accessions were evaluated using the previously described hydroponic setup [41]. After two days in the dark on the medical gauze of the germination device, uniform and robust rapeseed seeds were exposed to light (180 μmol photons m^−2^s^−1^) and grew for four days in a greenhouse (60–80 percent relative humidity) under 16/8 h day/night cycles at 24 °C [70]. The Hoagland’s solution (the concentration of N was 15 Mm; control treatment) was composed of: 5 mM Ca (NO_3_)_2_·4 H_2_O, 5 mM KNO_3_, 2 mM MgSO_4_ ·7H_2_O, 1mM KH_2_PO_4_, 0.05 mM EDTA-Fe, 46 µM H_3_BO_3_, 9.14 µM MnCl_2_·4H_2_O, 0.77 µM ZnSO_4_·7H_2_O, 0.37µM NaMoO_4_·2H_2_O, and 0.32 µM CuSO_4·_5H_2_O. Six days after planting, seedlings were shifted to a growth device containing a quarter of Hoagland’s solution (two treatments, control and LN), as described by [43]. Each basin had 30 seedlings of five different lines (five seedlings for each line). Once a week, the nutrient solution was replaced. Each week, the 1/4 solution was changed to a 1/2 solution, and then 100% solution until harvesting. The N content was decreased to 0.5 mM for LN treatment by lowering KNO_3_ and replacing Ca(NO_3_)_2_ with CaCl_2_. A completely randomized design was applied to three independent hydroponic culture trials conducted at Oil Crops Research Institute, Chinese Academy of Agricultural Sciences-Wuhan, China.

### 4.2. Phenotypic Investigation

Three plants from each genotype were collected during harvest, and each plant was divided into root and shoot sections. Five root morphology traits (RMT) viz. total root length (TRL), total root surface area (TSA), total root volume (TRV), and total root number (TRN) were captured through images using a scanner (EPSON V700, Japan) and further analyzed by WinRHIZO software (Pro 2012b, Canada), while primary root length (PRL) was measured manually using a ruler. Eight biomass-related traits (BT), including root fresh weight (RFW) and shoot fresh weight (SFW), were measured manually by using a weighing balance. Root dry weight (RDW) and shoot dry weight (SDW) were measured after oven drying at 80 °C until a consistent weight was reached. Total dry weight (TDW) and total fresh weight (TFW) were estimated as SDW + RDW and SFW + RFW, respectively. The ratio of root-to-shoot fresh weight (RSRF) and ratio of root-to-shoot dry weight (RSRD) were measured as the ratio between RFW and SFW and ratio between RDW and SDW, respectively.

### 4.3. Statistical Analysis

Statistical analysis was conducted using BLUE values for 13 traits studied under nitrogen stress across three trials. Statistically significant differences between treatments were estimated using a paired samples t-test, with α = 0.05 as a significant threshold. Basic statistics and broad-sense heritability were calculated using QTL Ici mapping 4.2 [36,71]. The “PerformanceAnalytics” package in R software was used to calculate Pearson correlation at a significance level of (*p* < 0.05). The response of each trait to LN was represented by the increase or decrease of LN relative to CK, calculated as (LN-CK)/LN × 100%.

### 4.4. Association Analysis

Using the new *B. napus* 50 K Illumina Infinium SNP array, 327 *B. napus* lines were genotyped. After filtering, there were 20,131 SNP markers for further investigation [25]. The trait–SNP association was investigated utilizing Best Linear Unbiased Estimates (BLUE) values of three LN trials via Tassel 5.0 software using a mixed linear model (MLM) with (Q + K) matrix [72]. To find marker–trait associations, an arbitrary cutoff value of 1/20,131 SNPs (−log _10_ (*p*) = 4.30) was used. The Manhattan and Quantile–Quantile (Q-Q) plots were generated using the qqman and ggplot2 tools, respectively [73,74]. The four-gamete criterion was used to identify marker haplotypes at each linked locus using Haploview software [75].

### 4.5. Candidate Gene Prediction

The complete gene list in the QTL cluster region was scanned using the *B. napus* “Darmor” reference genome information [76]. Potential candidate genes for nitrogen efficiency/LN nitrogen tolerance were identified using gene ontology (GO terms) from the TAIR website and gene functions recovered from prior studies [76].

### 4.6. Transcriptome Sequencing and Analysis

Based on the differences in SFW, 20 accessions (S5, S39, S46, S49, S64, S78, S118, S129, S140, S170, S189, S226, S251, S275, S283, S289, S291, S303, S313, and S324) with extremely high and 20 accessions (S18, S32, S90, S104, S106, S124, S145, S149, S161, S176, S193, S197, S205, S237, S252, S256, S265, S272, and S326) with extremely low SFW were selected from the association panel. The phenotypic values divided these accessions mainly into high nitrogen efficiency (HN) and low nitrogen efficiency (LN). These accessions were grown hydroponically under control and LN treatment conditions, and the same protocol of Hoagland’s solution was applied for the transcriptome experiment as described above. Samples were collected at two time points, 7 and 14 days after transplanting (T1 and T2).

Total RNA was extracted from root tissue of high-N and low-N efficiency accessions. Then, equal amounts of total RNA from 20 high-N and 20 low-N efficiency accessions were separately pooled. Three biological replicates, each obtained from three independent plants, were collected for RNA sequencing (RNA-seq) for each sample.

Twenty-four RNA-seq libraries (one tissue × two groups × two treatments × two-time points × three biological replicates per sample) were prepared for total RNA extraction with IRIzol reagent (Invitrogen, USA). An Illumina HiseqTM 2500 platform was used by Oebiotech Company in Shanghai, China, to construct sequencing libraries and conduct Illumina sequencing. Raw readings with 150 paired-end base pairs (bp) were filtered and aligned [77].

The clean reads were mapped using HISAT v2.0.4 and the *B. napus* ZS11 reference genome (https://www.genoscope.cns.fr/brassicanapus/data/, accessed on 22 April 2022) [76]. The WGCNA was conducted using the WGCNA package in R [78]. The “DESeq” R package was utilized to identify DEGs using ≤ 0.05 for the false discovery rate (FDR) and |log2 ratio| ≥ 1 as criteria.

### 4.7. Validation of DEGs by Quantitative Real-Time Polymerase Chain Reaction (qRT-PCR)

Twelve differentially expressed candidate genes were assessed by qRT-PCR to measure the reliability of the RNA-seq data, as previously described [79]. The primer sequences are presented in Table S8. The SYBR qPCR Master Mix (Vazyme) was used with the CFX96 for qRT-PCR analysis (BIO-RAD). Each sample was subjected to three technical replications. The 2^−ΔΔCT^ method was utilized to determine the relative expression of target genes using *B. napus* ACTIN2 as an internal control. [80].

## Figures and Tables

**Table 1:** Description of the 13 examined traits.

Classification	Trait Description	Abbreviations	Units
Root morphological traits (MT)	Primary root length	PRL	cm
	Total root length	TRL	cm
	Total root surface area	TSA	cm^2^
	Total root volume	TRV	cm^3^
	Total number of roots	TNR	number
Biomass-related traits (BM)	Shoot fresh weight	SFW	g
	Root fresh weight	RFW	g
	Shoot dry weight	SDW	g
	Root dry weight	RDW	g
	Total fresh weight	TFW	g
	Total dry weight	TDW	g
	Fresh root–shoot ratio	RSRF	RFW/SFW
	Dry root–shoot ratio	RSRD	RDW/SDW

**Table 2:** Descriptive statistics for investigated traits under control and low nitrogen treatment in association panel.

Trait	Control					LN					Control × N Stress	N Stress Impact (%)
	Min	Max	Mean	CV (%)	*h* ^2^	Min	Max	Mean	CV (%)	*h* ^2^		
PRL (cm)	12.2	34.3	24.3	14.5	0.62	16.3	36.5	25.9	12.8	0.58	**	6.4
TRL (cm)	434.3	1435.7	843.8	20.7	0.66	330.7	2104.9	912.1	21.6	0.58	*	7.5
TSA (cm^2^)	18.4	99.9	56.5	23.6	0.62	24.6	158.0	65.8	23.1	0.55	*	14.1
TRV (cm^3^)	0.062	0.604	0.311	29.9	0.57	0.154	0.719	0.319	25.4	0.53	ns	2.4
TRN	673.5	4804.5	1654.2	45.6	0.55	667	6213.9	2166.8	31.9	0.53	**	23.7
SFW (g)	1.291	4.889	3.243	20.1	0.72	0.858	4.062	1.799	18.7	0.66	**	−80.3
SDW (g)	0.234	1.377	0.741	26.5	-	0.266	0.992	0.594	20.8	-	**	−24.7
RFW (g)	0.261	0.788	0.503	19.2	0.60	0.227	1.479	0.538	22.2	0.60	**	6.4
RDW (g)	0.013	0.134	0.070	28.2	-	0.033	0.165	0.081	23.4	-	**	14.2
TFW (g)	1.617	5.599	3.746	19.3	0.70	1.077	5.541	2.345	18.3	0.65	**	−59.7
TDW (g)	0.277	1.476	0.809	25.9	-	0.302	1.157	0.676	19.6	-	**	−19.6
RSRF	0.102	0.248	0.159	15.9	0.70	0.199	0.470	0.311	16.1	0.55	**	48.9
RSRD	0.031	0.298	0.095	27.5	-	0.046	0.250	0.143	23.5	-	**	33.8

CV is coefficient of variation; *h*^2^ represents heritability; ns, not significant *, *p* < 0.05; **, *p* < 0.01, significance based on the analysis of variance.

**Table 3:** List of important QTL clusters for investigated traits under LN stress in association panel.

QTL Cluster	No. of SNPs	Peak SNP	Chr.	Position	PVE (%)	−log_10_P	Haplotype Block (Mb)	Traits
RT-A02-1	4	Bn-A02-p18994312	A02	17,997,697	7.0	4.5	17.95–18.25	RSRD
RT-A06-1	8	seq-new-rs31601	A06	7,556,204	10.5	6.8	7.25–7.72	TRN
RT-A09-1	19	Bn-A09-p1552993	A09	2,375,212	9.9	6.2	2.18–2.51	TRL, TSA, RFW, RDW, TRV, TFW, SFW
RT-A09-2	9	seq-new-rs41996	A09	4,405,703	21.2	12.7	4.35–4.41	TRL, TFW, RFW, TSA, SFW, TRV, RDW
RT-A10-1	4	Bn-A10-p11396195	A10	12,709,829	8.9	5.5	12.67–12.72	RFW, TFW, TRL, SFW
RT-A10-2	1	Bn-A10-p13659996	A10	13,700,523	10.0	6.5	13.56–13.70	TRN
RT-C03-1	2	seq-new-rs49231	C03	4,066,558	7.0	4.6	4.00–4.18	TFW, SFW
RT-C03-2	3	seq-new-rs28219	C03	8,417,394	8.4	5.5	8.32–8.41	TSA, TRL, TRV
RT-C03-3	2	seq-new-rs39672	C03	21,177,143	10.0	6.5	20.85–21.30	TRN
RT-C03-4	11	seq-new-rs41373	C03	27,421,341	9.0	5.8	27.42–27.46	TRV, TSA, TFW, TRL, RFW, SFW
RT-C06-1	2	seq-new-rs23016	C06	12,887,745	7.1	4.6	12.80–13.08	TDW, SDW
RT-C07-1	7	seq-new-rs46512	C07	35,123,112	20.4	12.8	35.12–35.17	RFW, TSA, TFW, TRL, SFW, TRV, RDW
RT-C08-1	4	seq-new-rs29850	C08	21,323,655	5.7	4.5	21.23–21.32	TRV, TSA, TFW
RT-C09-1	5	seq-new-rs34959	C09	34,541,392	11.9	7.4	34.38–34.54	TSA, TRL, RFW, TFW, SFW

**Table 4:** List of candidate genes identified through the integration of GWAS, WGCNA, differential expression analysis, and their FPKM values.

Hub Genes in the MEsalmon Module (GWAS + WGCNA)											
**Darmor_ID**	**HN-T1**	**HN-T2**	**HNCK-T1**	**HNCK-T2**	**LN-T1**	**LN-T2**	**LNCK-T1**	**LNCK-T2**	**QTN/QTL Cluster**	**Distance from Lead SNP (Kb)**	**Description**
BnaA09g04260D	7.22	27.94	9.28	37.08	7.39	25.75	6.49	34.92	RT-A09-1	273.55	Sugar transport protein 13
BnaA09g05270D	4.92	1.84	5.39	1.88	7.99	2.68	7.09	3.29	RT-A09-1	−205.92	Probable carboxylesterase 18
Hub genes in the MEbrown module (GWAS+WGCNA)											
BnaA09g08440D	1.93	4.34	1.84	3.41	2.12	4.73	1.70	3.23	RT-A09-2	249.99	BTB/POZ domain-containing protein
BnaC07g30970D	13.56	13.81	15.98	12.58	15.33	13.72	18.75	14.97	RT-C07-1	−118.79	Exocyst complex component
Significant DEGs from GWAS + RNA-sequencing data											
BnaA09g04280D	0.72	0.65	0.55	0.68	1.69	1.43	1.10	2.25	RT-A09-1	264.71	TRAF-like family protein
BnaA09g08450D	9.19	13.66	11.39	15.52	12.12	12.26	9.48	12.54	RT-A09-2	241.43	MVB pathway protein
BnaA10g16560D	0.00	0.08	0.08	3.80	7.23	0.23	0.00	0.00	RT-A10-1	147.27	/
BnaA10g17620D	5.56	6.81	6.50	6.40	4.37	3.03	4.04	4.40	RT-A10-1	−268.85	hydrolase II (PTH2) family protein
BnaA10g19550D	3.06	3.46	2.79	4.28	5.05	3.37	2.45	3.75	RT-A10-2	−150.98	unknown protein
BnaA10g19700D	46.34	27.83	38.00	18.89	16.09	12.86	18.94	12.44	RT-A10-2	−202.00	xyloglucan hydrolase 5 (XTH5)
BnaC03g34740D	1.22	1.04	1.49	0.92	1.15	0.96	1.05	1.08	RT-C03-3	105.06	GNS1/SUR4 membrane protein
BnaC03g42900D	16.87	15.62	28.99	14.44	26.24	17.19	27.69	21.44	RT-C03-4	−217.19	lipid-transfer protein
BnaC06g11180D	1.10	1.63	1.28	1.72	0.06	0.11	1.42	0.10	RT-C06-1	−262.51	/
BnaC06g11230D	0.81	0.50	1.57	1.02	0.83	1.10	1.22	1.01	RT-C06-1	−280.51	nicotinamidase 1 (NIC1)
BnaC07g30250D	2.51	1.76	2.50	1.18	1.07	0.60	0.89	0.43	RT-C07-1	295.55	/
BnaC07g30400D	5.52	3.82	10.18	8.55	3.83	5.54	9.20	10.00	RT-C07-1	223.14	SLAC1 homologue 3 (SLAH3)

## Data Availability

The datasets generated or analyzed during the present study are available from the corresponding authors on reasonable request.

## Supplementary Materials

The following supporting information can be downloaded at: https://www.mdpi.com/article/10.3390/ijms23147958/s1.
